# MAGE-TRIM28 complex promotes the Warburg effect and hepatocellular carcinoma progression by targeting FBP1 for degradation

**DOI:** 10.1038/oncsis.2017.21

**Published:** 2017-04-10

**Authors:** X Jin, Y Pan, L Wang, L Zhang, R Ravichandran, P R Potts, J Jiang, H Wu, H Huang

**Affiliations:** 1 Department of Digestive Surgical Oncology, Union Hospital, Tongji Medical College, Huazhong University of Science and Technology, Wuhan, China; 2 Department of Biochemistry and Molecular Biology, Mayo Clinic College of Medicine, Rochester, MN, USA; 3 Department of Medical Informatics and Statistics, Mayo Clinic College of Medicine, Rochester, MN, USA; 4 Department of Laboratory Medicine and Pathology, Mayo Clinic College of Medicine, Rochester, MN, USA; 5 Department of Cell and Molecular Biology, St. Jude Children’s Research Hospital, Memphis, TN, USA; 6 Department of Tumor Biological Treatment, The Third Affiliated Hospital of Soochow University, Changzhou, China; 7 Department of Pancreatic Surgery, Union Hospital, Tongji Medical College, Huazhong University of Science and Technology, Wuhan, China; 8 Department of Urology, Mayo Clinic College of Medicine, Rochester, MN, USA; 9 Mayo Clinic Cancer Center, Mayo Clinic College of Medicine, Rochester, MN, USA

## Abstract

Hepatocellular carcinoma (HCC) is one of the leading cause of cancer death in the world. Fructose-1,6-biphosphatase (FBP1), a rate-limiting enzyme in gluconeogenesis, has been identified recently as a tumor suppressor in HCC and other cancer types. In this study, we demonstrated that the tripartite motif-containing protein 28 (TRIM28) binds directly to and promotes FBP1 for ubiquitination and degradation. MAGE-A3 and MAGE-C2, which are known to be overexpressed in HCC, can enhance TRIM28-dependent degradation of FBP1 by forming ubiquitin ligase complexes with TRIM28. We further showed that expression of TRIM28 increased glucose consumption and lactate production by promoting FBP1 degradation in HCC cells and that FBP1 is a key mediator of TRIM28-induced HCC growth in culture and in mice. Moreover, we demonstrated that FBP1 and TRIM28 protein levels inversely correlated in HCC patient specimens. Finally, we showed that the proteasome inhibitor bortezomib mitigated the Warburg effect by inhibiting FBP1 degradation in HCC. Collectively, our findings not only identify oncogenic MAGE-TRIM28 complex-mediated proteasome degradation of FBP1 as a key mechanism underlying downregulation of FBP1 proteins in HCC, but also reveal that MAGE-TRIM28-regulated reprogramming of cancer cell metabolism and HCC tumorigenesis is mediated, at least in part, through FBP1 degradation.

## Introduction

Hepatocellular carcinoma (HCC) is the fourth leading cause of cancer-related death in China and is a common cancer type worldwide. For patients with well-defined tumors, hepatic resection and liver transplantation represent two best medical interventions, but the 5-year survival rate still remain slow (approximately 60–70%).^1^ It is very common that surgery is no longer suitable for HCC patients because of the fact that tumors are usually at late stage when diagnosed. Unfortunately, very few therapy options are currently available for effective treatment of advanced HCC.^1^ Thus, novel therapeutics is urgently needed for effective treatment of HCC.

The Warburg hypothesis postulates that tumor cells metabolize glucose to lactate even when oxygen is abundant. Increased aerobic glycolysis is a common characteristic in many human cancers including HCC. HCC cell proliferation is shown to correlate with glucose metabolism.^2^ Investigation of the molecular mechanism of glucose metabolism could lead to the development of new treatment for HCC.

Deregulation of oncogenes and tumor-suppressor genes is demonstrated to be responsible to the Warburg effect in HCC.^3^ Gluconeogenesisis a key factor influencing aerobic glycolysis. Fructose-1,6-biphosphatase (FBP1) is a rate-limiting enzyme in gluconeogenesis by converting fructose-1,6-bisphosphate to fructose-6-phosphate.^4^ *FBP1* and *FBP2* are two human FBPase genes.^5^ *FBP1* consists of seven exons, and encodes a 362-amino-acid protein, primarily expressed in the liver.^6^ In agreement with the key role of FBP1 in modulating glucose metabolism in cancer, decreased expression of FBP1 associates with HCC development and progression,^7^ although the exact mechanism underlying FBP1 downregulation in HCC is not fully understood.

The family of the tripartite motif-containing proteins (TRIM) consists of 60 members. Each member shares similar domains, which include a RING domain, one or two cysteine/histidine-rich motifs or called B-box domains, and a coiled-coil domain.^8^ TRIM28 functions as a corepressor of Kruppel-associated box zinc-finger factors. TRIM28 also acts as an E3 ubiquitin ligase and forms MAGE-TRIM28 E3 ubiquitin ligase complexes in cancer to target tumor-suppressor proteins such as 5' adenosine monophosphate-activated protein kinase (AMPK) and p53 for ubiquitination and proteasome degradation.^9, 10^

In this study, we demonstrated that the MAGE-TRIM28 E3 ubiquitin ligase complex promotes FBP1 protein for degradation in HCC cells. We further demonstrated that the oncogenic MAGE-TRIM28 complexes regulate glucose metabolism in HCC cells and this effect is mediated at least in part through FBP1 degradation. Moreover, we showed that bortezomib, a potent and reversible proteasome inhibitor, enables to overcome the Warburg effect in HCC by inhibiting FBP1 degradation.

## Results

### TRIM28 interacts with FBP1 in HCC cells

**Figure 1:**
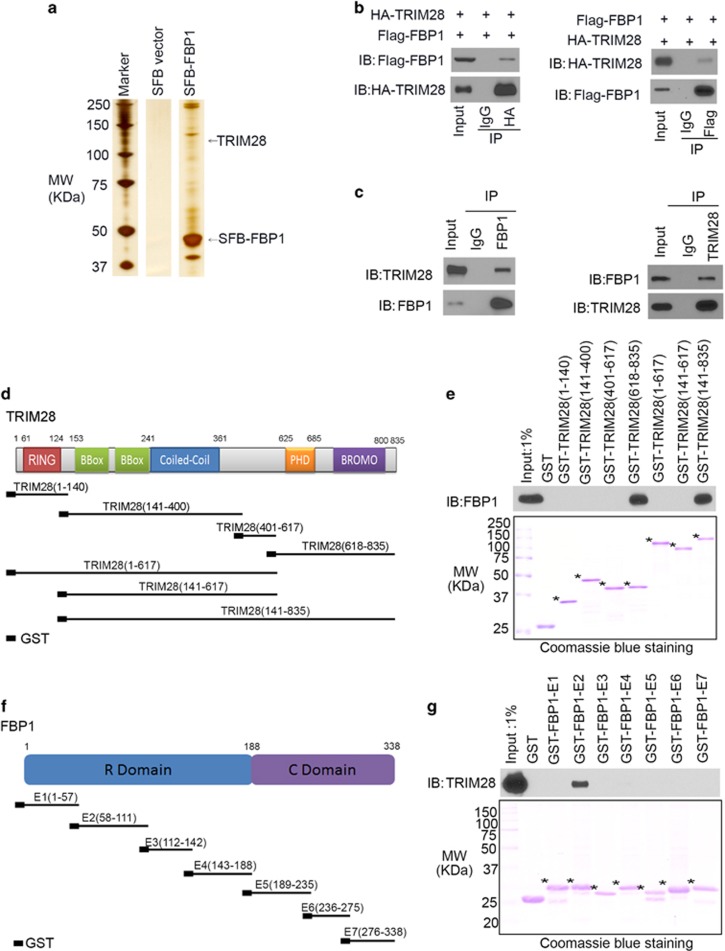
TRIM28 interacts with FBP1 in HCC cells. (**a**) Sodium dodecyl sulfate–polyacrylamide gel electrophoresis (SDS–PAGE) and silver staining of proteins purified by tandem affinity purification from 293T cells transfected with control vector or SFB-tagged FBP1. (**b**) Western blot analysis of reciprocal co-immunoprecipitation of ectopically expressed Flag-FBP1 and HA-TRIM28 in 293T cells. (**c**) Western blot analysis of reciprocal co-immunoprecipitation of endogenous FBP1 and TRIM28 proteins in HepG2 cells. (**d**) Schematic diagram depicting a set of GST-TRIM28 recombinant protein constructs. (**e**) Western blot analysis of FBP1 proteins in HepG2 whole-cell lysate pulled down by GST or GST-TRIM28 recombinant proteins. (**f**) Schematic diagram depicting a set of GST-FBP1 recombinant protein constructs. (**g**) Western blot analysis of TRIM28 proteins in HepG2 whole-cell lysate pulled down by GST or GST-FBP1 recombinant proteins.

To explore the regulatory mechanisms of FBP1 functions, we constructed a FBP1 mammalian expression vector (SFB-FBP1) containing S, Flag and biotin-binding protein (streptavidin) binding peptide tags to identify cellular proteins associated with FBP1. SFB-FBP1 and the backbone vector were transfected separately into 293T cells and cell extracts were prepared for tandem affinity purification coupled mass spectrometry. A large number of new binding partners such as TRIM28 were identified (Figure 1a). The interaction between ectopically expressed Flag-FBP1 and HA-TRIM28 in 293T cells and endogenous FBP1 and TRIM28 in HepG2 HCC cells were confirmed by reciprocal co-immunoprecipitation assays (Figures 1b and c). As *TRIM28* mRNA level in liver tumors was higher than that in normal liver tissues (Supplementary Figure S1a),^11^ we chose to further investigate the molecular basis of the interaction between TRIM28 and FBP1 and the biological significance of their interaction.

TRIM28 is a multi-domain protein with multiple functions^12^ (Figure 1d). To determine which region(s) in TRIM28 are involved in FBP1 binding, we generated seven glutathione *S*-transferase (GST)-TRIM28 recombinant proteins as reported previously^12^ (Figure 1d). GST pull-down assays revealed that the region containing the amino acids (aa) 617–835 bound to FBP1 protein in HepG2 cell lysate (Figure 1e), suggesting that PHD and BROMO domains in the COOH-terminal half mediate the binding with FBP1. To define which region(s) in FBP1 mediate its interaction with TRIM28, we constructed seven GST-FBP1 recombinant proteins corresponding to seven exons of FBP1 as reported previously^7^ (Figure 1f). GST pull-down assays demonstrated that GST-FBP1 E2 (aa 58–111), but not GST and other GST-FBP1 recombinant proteins, interacted with TRIM28 (Figure 1g). Although FBP1 is mainly expressed in the cytoplasm and functions as one of the rate-limiting enzyme in gluconeogenesis, a portion of FBP1 proteins is also expressed in the nucleus and acts to inhibit the activity of nuclear factors such as hypoxia-inducible transcription factor via direct protein–protein interaction.^7^ We further showed that FBP1 interacted with TRIM28 both in the cytosol and nucleus in HepG2 cells (Supplementary Figures S1b and c). These data indicate that TRIM28 interacts with FBP1 in HCC cells.

### FBP1 is a degradation substrate of E3 ubiquitin ligase TRIM28

**Figure 2:**
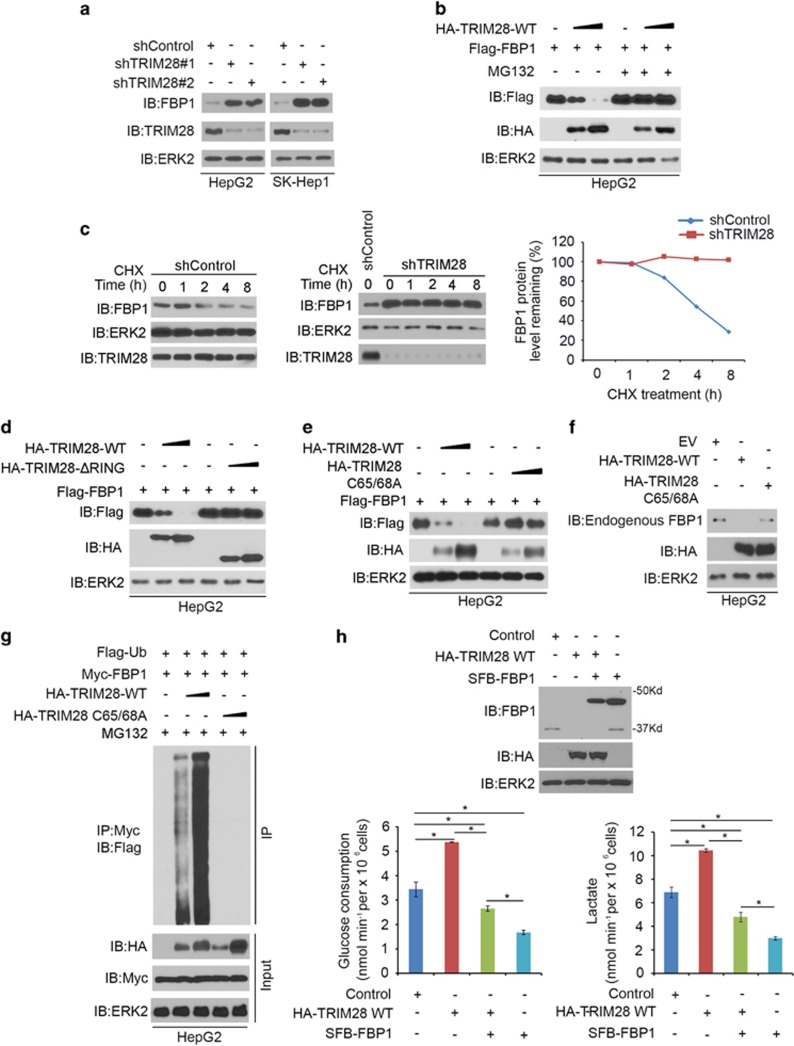
FBP1 is a degradation substrate of E3 ubiquitin ligase TRIM28. (**a**) HepG2 and SK-Hep-1 cells were infected with control or two independent TRIM28-specific shRNAs. Cells were harvested for western blot analysis after 48 h transfection. (**b**) Western blot analysis of whole-cell lysate of HepG2 cells transfected with the indicated constructs. Cells were treated with or without 20 μM of MG132 for 8 h before harvest. (**c**) HepG2 cells were infected with control and TRIM28-specific shRNAs. After 48 h, cells were treated with 50 μg/μl cycloheximide (CHX). At different time points, cells were harvested for western blot analysis. At each time point, the intensity of FBP1 was normalized to the intensity of ERK2 (loading control) first and then to the value at the 0-h time point. (**d**) HepG2 cells were transfected with indicated plasmids for 24 h followed by western blot analysis. (**e**) Western blot analysis of whole-cell lysate of HepG2 cells transfected with the indicated constructs. (**f**) Western blot analysis of whole-cell lysate of HepG2 cells transfected with the indicated constructs. (**g**) Western blot analysis of whole-cell lysate of HepG2 cells transfected with the indicated constructs. Cells were treated with or without 20 μM of MG132 for 8 h before harvest. (**h**) Measurement of glucose consumption and l-lactate production in spent medium of HepG2 cells 48 h after transfected with indicated constructs and western blot analysis of whole-cell lysate of HepG2 cells. *P< 0.01.

We systematically investigated whether TRIM28 regulates FBP1 protein in HCC cells. Knockdown of TRIM28 resulted in an increase in FBP1 protein level in HepG2 and SK-Hep-1HCC cell lines (Figure 2a). It has been reported previously that TRIM28 possess an E3 ligase function.^13, 14^ Overexpression of HA-TRIM28 decreased the level of ectopically expressed Flag-FBP1 protein in a dose-dependent manner in HepG2 cells, and this effect of TRIM28 was completely blocked by treatment of cells with the proteasome inhibitor MG132 (Figure 2b). In agreement with these findings, neither knockdown nor overexpression of TRIM28 has any overt effect on *FBP1* mRNA level in HepG2 cells (Supplementary Figure S1d). Knockdown of endogenous TRIM28 prolonged endogenous FBP1 protein half-life in HepG2 cells (Figure 2c). The RING domain of TRIM28 is required for its E3 ligase activity. RING domain deletion or C65/68A point mutants of TRIM28 lack E3 ligase activity.^9^ Enzymatic dead mutant of TRIM28 was unable to promote polyubiquitination and degradation of FBP1 (Figures 2d–g). It is worth noting that lysine residue(s) for ubiquitination in the known degradation substrates of TRIM28, including AMPK, p53, IRF7 and NPM1/B23,^9, 10, 12, 13^ have not been identified. Therefore, further investigation of the lysine residue(s) critical for TRIM28-mediated ubiquitination and degradation of FBP1 and the known substrates is warranted.

As FBP1 is a key enzyme in gluconeogenesis that antagonizes the glycolysis process,^7^ we found that knocking down FBP1 increased glucose consumption and lactate production in HepG2 cells (Supplementary Figure S1e). The effect of FBP1 is specific as restored expression of short hairpin RNA (shRNA)-resistant FBP1 reversed FBP1 knockdown-induced increase in glucose consumption and lactate production (Supplementary Figure S1e). We next sought to determine whether the TRIM28 E3 ligase affects glucose metabolism by regulating FBP1 degradation in HCC cells. Overexpression of wild-type TRIM28 increased glucose consumption and lactate production in HepG2 cells. Importantly, the effect of TRIM28 was largely inhibited by co-expression of FBP1 (Figure 2h). It is worth noting that knockdown of TRIM28 in HeLa cells increased glucose consumption and this effect is likely mediated in part through AMPK degradation.^10^ Intriguingly, although AMPK generally acts as an inhibitor of the Warburg effect,^15^ acute activation of AMPK has been shown to promote glucose consumption,^16^ which is consistent with the findings reported recently by Pineda *et al.*^10^ We further showed that knockdown of TRIM28 resulted in a much greater increase in FBP1 protein than AMPK in both HepG2 and SK-Hep1 cell lines (Supplementary Figures S1f and g). These findings suggest that higher level of TRIM28 increases glucose consumption by primarily decreasing FBP1 levels in HCC cells. Thus, our data not only identify TRIM28 as an E3 ligase of FBP1, but also suggest that TRIM28-regulated reprogramming of cancer cell metabolism is mediated, at least in part, through FBP1 degradation in HCC cells.

### MAGE family proteins enhance FBP1 degradation by forming E3 ligase complexes with TRIM28

**Figure 3:**
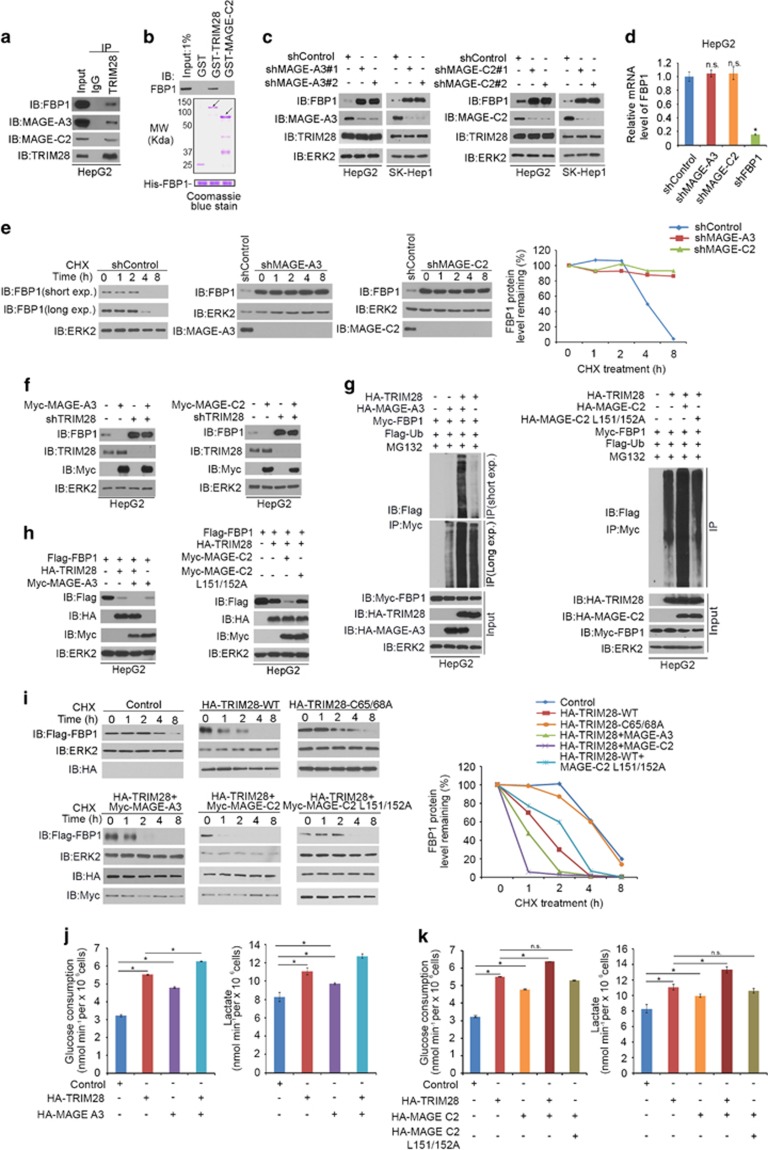
MAGE family proteins enhance FBP1 degradation by forming E3 ligase complexes with TRIM28. (**a**) HepG2 cell lysates were prepared for co-immunoprecipitation with anti-TRIM28 antibody followed by western blot analysis. (**b**) Bacterially expressed His-FBP1 proteins and GST, GST-TRIM28 or GST-MAGE-C2 recombinant proteins were subjected to *in vitro* protein binding assays followed by western blot analysis. Input samples were analyzed by Coomassie blue staining. (**c**, **d**) HepG2 and SK-Hep-1 cells were infected with control or two independent MAGE-A3 or MAGE-C2-specific shRNAs. After 48 h, cells were harvested for western blot (**c**) and reverse transcriptase (RT)–quantitative PCR (qPCR) analysis (**d**). (**e**) HepG2 cells were infected with control and MAGE-A3 and MAGE-C2 specific shRNAs. After 48 h, cells were treated with 50 μg/ml cycloheximide (CHX). At different time points, cells were harvested for western blot analysis. Exp., exposure. (**f**) HepG2 cells were infected with control and TRIM28-specific shRNAs. After 24 h, HepG2 cells were transfected with indicated constructs for another 24 h followed by western blot analysis. (**g**) HepG2 cells were transfected with indicated constructs for 24 h followed by western blot analysis. (**h**) HepG2 cells were transfected with indicated constructs for 20 h followed by treatment with 20 μM of MG132 for 8 h. Immunoprecipitated Myc-FBP1 proteins were analyzed by western blot. (**i**) HepG2 cells were transfected with control or indicated plasmids. After 24 h, cells were treated with 50 μg/μl CHX. At indicated time points, cells were harvested for western blot analysis. (**j**, **k**) Glucose consumption and l-lactate production were measured in the spent medium of HepG2 cells 48 h after transfected with indicated constructs. *P<0.01; NS, not significant.

It had been reported previously that MAGE proteins are key components of some specific E3 ubiquitin ligases.^9, 10, 17, 18^ MAGE-A3/6 and MAGE-C2 specifically bind to the TRIM28.^9, 10^ MAGE-TRIM28 E3 ligase complexes can induce degradation of p53 and AMPK proteins via ubiquitination.^10, 19, 20, 21^ We examined whether MAGE family proteins have any role in TRIM28-mediated degradation of FBP1. Co-immunoprecipitation assays revealed that similar to FBP1, endogenous MAGE-A3 and MAGE-C2 proteins were immunoprecipitated by anti-TRIM28 antibody from HepG2 cell lysate (Figure 3a). *In vitro* protein-binding assay using recombinant proteins purified from bacteria showed that His-FBP1 was only pulled down by GST-TRIM28 but not GST-MAGE-C2, indicating that FBP1 binds directly to TRIM28, but not MAGE-C2 (Figure 3b). Knockdown of endogenous MAGE-A3 and MAGE-C2 increased FBP1 protein level and prolonged the half-life of endogenous FBP1 protein without any overt effect on *FBP1* mRNA expression in HepG2 and SK-Hep-1 cells (Figures 3c–e). Conversely, overexpression of Myc-MAGE-A3 and Myc-MAGE-C2 induced degradation of ectopically expressed Flag-FBP1, whereas these effects were abolished by knockdown of TRIM28 (Figure 3f). Furthermore, overexpression of Myc-MAGE-A3 and Myc-MAGE-C2 enhanced FBP1 polyubiquitination and degradation mediated by HA-TRIM28 and shortened the half-life of FBP1 protein (Figures 3g–i). Accordingly, expression of these proteins also resulted in increased glucose consumption and lactate level in HepG2 cells (Figures 3j and k). It has been reported previously that L151/152A mutants of MAGE-C2 cannot bind to TRIM28.^12^ We demonstrated that Myc-MAGE-C2 L151/152A failed to enhance TRIM28-mediated FBP1 polyubiquitination and degradation (Figures 3g and h). These results indicate that MAGE family proteins enhance TRIM28-mediated degradation of FBP1. These findings and others in the literature suggest that the effect of TRIM28 on glucose metabolism is mediated in part by one of its degradation substrates including FBP1.

### FBP1 is a key mediator of TRIM28-regulated HCC growth in culture and in mice

**Figure 4:**
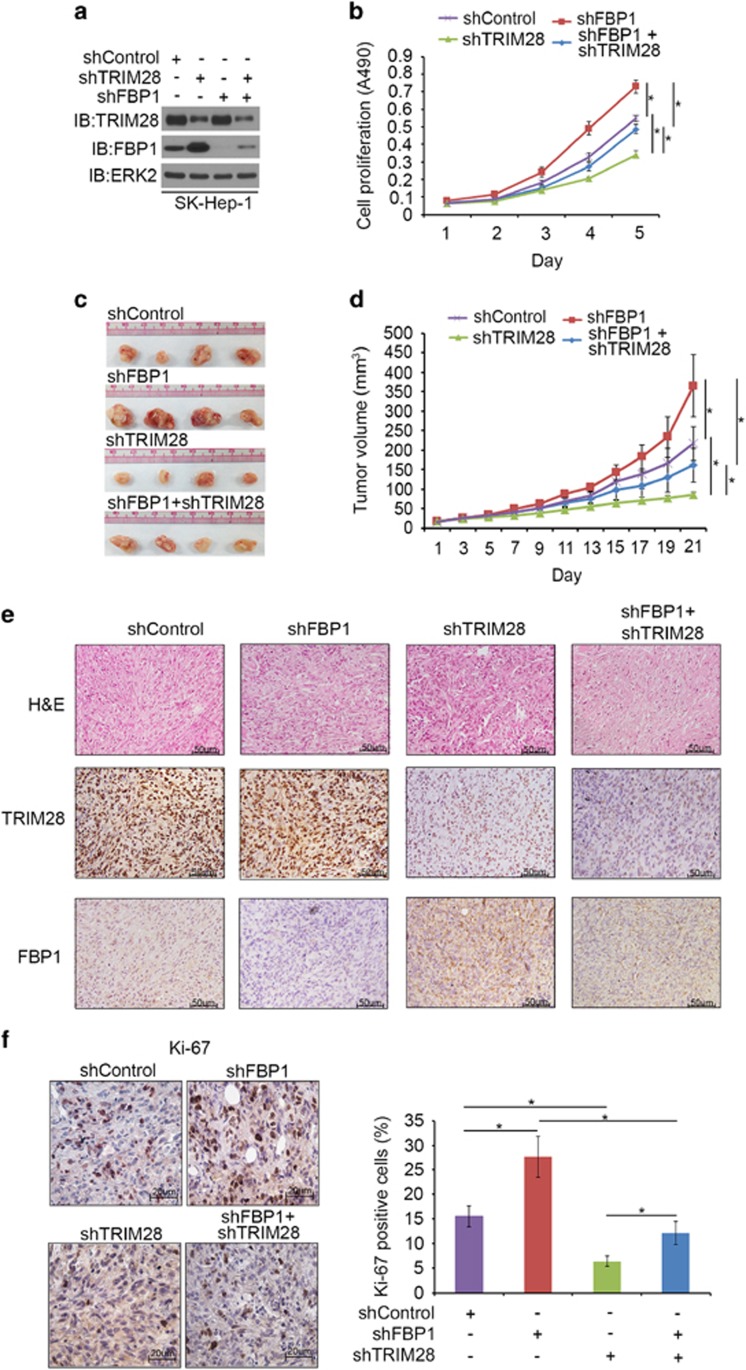
FBP1 is a key mediator of TRIM28-regulated HCC growth in culture and in mice. (**a**, **b**) SK-Hep-1 cells were infected with lentivirus expressing control or TRIM28 and/or FBP1-specific shRNAs. Cells were harvested for western blot analysis (**a**) and MTS assay (**b**) after 48-h infection. All data show mean values±s.d. (error bar) (*n*=6). *P< 0.01. (**c**, **d**) SK-Hep-1 cells were infected with lentivirus as in **a**. Seventy-two hours after infection, cells were administered into subcutaneous layer of the right flank of mice. Xenografts growth was measured every other day for 21 days, and tumors were harvested and photographed. Data present means + s.d. (*n*=4). *P<0.01. (**e**) Tumors harvested in **c** were sectioned for hematoxylin and eosin (H&E) and IHC of TRIM28 and FBP1 in xenografts tissues as indicated. (**f**) IHC analysis of Ki-67 expression in xenografts was performed and staining was quantified. All results shown are mean values± s.d. (error bar) (*n*=4). *P<0.01.

Previous studies show that FBP1 protein level is decreased in renal carcinoma cells and restoration of FBP1 protein level inhibits renal cancer cell proliferation.^7^ Next, we sought to determine the role of FBP1 protein in HCC cell growth. SK-Hep-1 cells were infected with control, FBP1- and/or TRIM28-specific shRNA and lentivirus-infected cells were used for western blot, MTS (3-(4,5-dimethylthiazol-2-yl)-5-(3-carboxymethoxyphenyl)-2-(4-sulfophenyl)-2H-tetrazolium) assay and animal studies. Knockdown of both FBP1 and TRIM28 was effective in SK-Hep-1 cells before injection (Figure 4a). MTS assay showed that knockdown of FBP1 increased cell growth (Figure 4b). In contrast, knockdown of TRIM28 alone decreased cell growth (Figures 4a and b). Importantly, co-knockdown of FBP1 largely attenuated TRIM28 knockdown-induced inhibition of cell growth (Figures 4a and b). Similarly, knockdown of TRIM28 alone inhibited growth of SK-Hep-1 xenografts in mice, but knockdown of FBP1 alone promoted xenograft growth (Figures 4c and d). However, double knockdown of FBP1 and TRIM28 abolished TRIM28 knockdown-induced inhibition of SK-Hep-1 tumor growth in mice (Figures 4c and d). The xenografts were subjected to hematoxylin and eosin and immunohistochemical (IHC) analysis for TRIM28, FBP1 and Ki-67 expression (Figures 4e and f). IHC analysis confirmed the effectiveness of TRIM28 and FBP1 knockdown in harvested xenograft tumors (Figure 4e). Knockdown of FBP1 resulted in an increase in Ki-67 staining compared with the control group, but knockdown of TRIM28 decreased Ki-67 staining in tumor tissues compared with control tumors (Figure 4f). However, double knockdown of FBP1 and TRIM28 had little effect on Ki-67 staining in these tumors compared with control knockdown (Figure 4f). These data suggest that FBP1 is a key mediator of TRIM28-regulated HCC cell growth *in vitro* and *in vivo*.

### FBP1 protein levels inversely correlate with TRIM28 expression in HCC patient specimens

**Figure 5:**
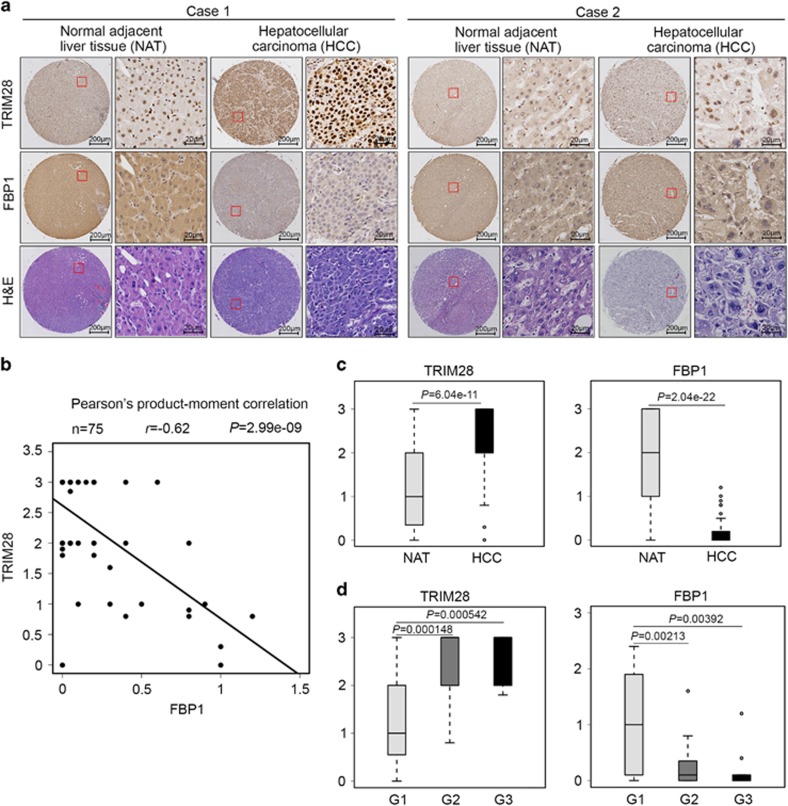
FBP1 protein levels inversely correlate with TRIM28 expression in HCC patient specimens. (**a**) Images of IHC analysis of FBP1 and TRIM28 protein expression and hematoxylin and eosin (H&E) staining on TMA (*n*=75) tissue sections. Scale bars are shown as indicated. (**b**) Correlation analysis of the staining index (SI) of expression levels of FBP1 and TRIM28 proteins in human HCC specimens (*n*=75). Pearson’s product-moment correlation co-efficiency and the *P*-values are also shown. (**c**) Box plots of TRIM28 and FBP1 protein expression based on their SI in nonmalignant adjacent tissues (NAT) and HCC specimens. The *P*-values are also shown. (**d**) Box plots of TRIM28 and FBP1 protein expression based on their SI in HCC specimens at different clinical stages. G1, G2 and G3 represent well-differentiated, moderately differentiated and poorly differentiated tumors, respectively. The *P*-values are also shown.

To investigate the clinical correlation of TRIM28 regulation of FBP1 protein level in HCC cell lines, we sought to determine the correlation between TRIM28 and FBP1 protein levels in human HCC specimens using tissue microarray (TMA). Specifically, we used a TMA containing 75 normal tumor paired specimens. IHC staining was evaluated by measuring both percentage of staining positive cells and staining intensity. Examples of both strong and weak staining of TRIM28 and FBP1 proteins and corresponding hematoxylin and eosin staining are shown in Figure 5a. FBP1 was inversely correlated with TRIM28 expression in this cohort (Pearson’s product-moment correlation *r*=−0.62, *P*=2.99e–09) (Figure 5b). Further analysis indicated that liver tumor tissues had higher TRIM28 expression level (*P*=6.04e–11) but lower level of FBP1 (*P*=2.04e–11) compared with adjacent normal liver tissues (Figure 5c). Moreover, it was shown that TRIM28 expression was increased with tumor stage and TRIM28 expression was positively associated with tumor stage. In contrast, tumor with lower stage had higher FBP1 expression level, which was negatively associated with tumor stage (Figure 5d). These data indicate that FBP1 and TRIM28 protein level inversely correlate in human HCC specimens.

### Bortezomib inhibits FBP1 degradation and regulates the Warburg effect in HCC

**Figure 6:**
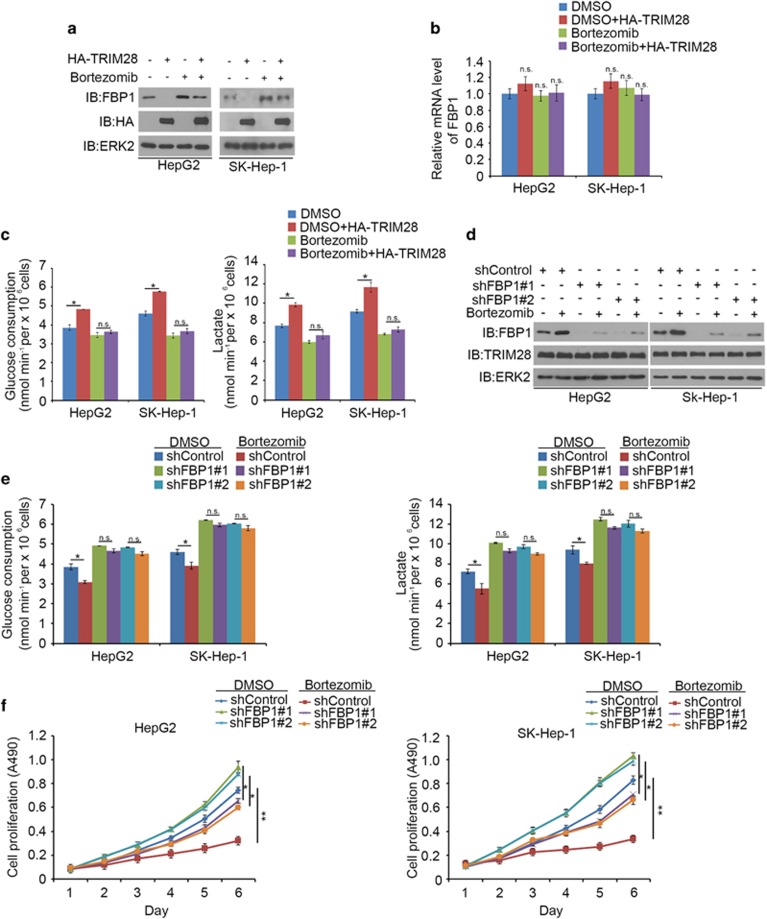
Bortezomib inhibits FBP1 degradation and regulates Warburg effect in HCC. (**a**-**c**) HepG2 cells were transfected with indicated constructs for 20 h followed by treatment with 80 nM of bortezomib for 8 h. Cells were then harvested for western blot analysis (**a**) and reverse transcriptase (RT)–quantitative PCR (qPCR) (**b**) and spent medium were used for measurement of glucose and lactate levels (**c**). *P< 0.01; NS, not significant. (**d**, **e**) HepG2 and SK-Hep-1 cells were infected with lentivirus-expressing control or FBP1-specific shRNAs. Twenty-four hours after infection, cells were treated with 40 nM of bortezomib for another 24 h, and the cells were harvested for western blot analysis (**d**) and spent medium was collected for measurement of glucose and l-lactate levels (**e**). *P< 0.01. (**f**) HepG2 and SK-Hep-1 cells were infected with lentivirus expressing control or FBP1-specific shRNAs. Forty-eight hours after infection, cells were treated with 20 nM of bortezomib for MTS assay. All data are mean values±s.d. (error bar) (*n*=6). *P <0.01; **P<0.001.

Bortezomib, a boronic acid dipeptide derivative, is a 26S proteasome inhibitor.^22^ It has been approved by Food and Drug Administration for multiple myeloma and mantle cell lymphoma treatment.^23, 24^ Bortezomib can inhibit HCC cell proliferation, migration and invasion.^25, 26^ The mechanisms of action of bortezomib in HCC cells are very complex and are not fully understood. We examined whether bortezomib inhibits TRIM28-mediated degradation of FBP1. TRIM28-induced decrease in FBP1 protein level was completely blocked by bortezomib treatment and had no overt effect on *FBP1* mRNA level in HepG2 and SK-Hep-1 cell lines (Figures 6a and b).The changes in glucose consumption and lactate levels were consistent with the FBP1 protein level alterations in both HepG2 and SK-Hep-1 cells treated with bortezomib (Figure 6c). Next, we sought to determine if bortezomib regulates proliferation of HCC cells via impairing FBP1 degradation. HepG2 and SK-Hep-1 cells were infected with control, FBP1-specific shRNA and treated with dimethylsulfoxide or bortezomib, respectively. Lentivirus-infected cells were used for western blot, MTS assay and glucose metabolism analysis. We demonstrated that bortezomib treatment increased FBP1 protein level, decreased glucose consumption and lactate level, and inhibited cell growth in both HepG2 and SK-Hep-1 cell lines (Figures 6d–f). Importantly, the effects of bortezomib on glucose consumption and lactate level and cell growth were largely diminished by knockdown of FBP1 by two independent shRNAs (Figures 6d–f). Together, these data indicate that bortezomib regulates the Warburg effect and this effect is mediated at least in part by modulating FBP1 protein levels in HCC cells.

## Discussion

Acting as a rate-limiting enzyme of gluconeogenesis, FBP1 promotes the conversion of fructose-1,6-bisphosphate to fructose-6-phosphate and inorganic phosphate, thereby antagonizing glycolysis. Increasing evidence suggests that FBP1 functions as a tumor suppressor. It is proposed that loss of FBP1 causes an increase in many processes including glucose uptake, formation of tetrameric PKM2, maintenance of ATP production and ultimately increased glycolysis. Furthermore, FBP1 directly binds to and inhibits hypoxia-inducible factors in the nucleus by mediating transcriptional downregulation of hypoxia-inducible factor target genes including *PDK1, LDHA* and *GLUT1*.^10^ Loss or downregulation of FBP1 is detected in several cancer types and decreased expression of FBP1 associates with tumor node metastasis stage and poor survival of patients.^3, 27, 28^ Restored FBP1 expression markedly suppresses cancer cell growth. Previous studies also show that FBP1 is epigenetically silenced in human cancers. Snail, nuclear factor-kappaB (NFκB) and lysine (K)-specific demethylase 1A (LSD1) have been shown to repress FBP1 expression by inducing *FBP1* promoter methylation.^4, 29, 30^ Apart from promoter methylation and copy-number loss responsible for the decreased *FBP1* mRNA expression,^3^ we provide experimental evidence demonstrating that the E3 ubiquitin ligase TRIM28 has a critical role in regulating FBP1 protein levels through a post-translational mechanism in HCC cells.

It has been reported that TRIM28 promotes proliferation and metastatic progression of breast cancer and associates with aggressive clinical features in gastric, non-small cell lung cancer and pancreatic cancer.^31, 32, 33^ Although MAGE proteins are normally expressed only in the testis, they are aberrantly re-expressed in cancerous tissues.^34, 35^ MAGE proteins bind with specific RING proteins to form MAGE-RING complexes in cells, including MAGE-C2-TRIM28, MAGE-A3/6-TRIM28 and MAGE-D1-Praja-1.^9^ Furthermore, each MAGE protein binds to a specific RING protein through the MHD domain. MAGE-A3/6 and MAGE-C2 proteins specifically bind with TRIM28.^9, 36^ Of note, MAGE-A3 and -C2 proteins are highly expressed in HCC.^37, 38, 39^ However, the mechanism of action of MAGEs and TRIM28 proteins in HCC tumors is not well understood. It has been demonstrated that MAGE proteins act as regulatory molecules to enhance the activity of E3 ubiquitin ligases. MAGE-C2 enhances TRIM28-induced p53 ubiquitination.^9, 36^ Oncogenic MAGE-A3/6-TRIM28 ubiquitin ligase inhibits autophagy by causing polyubiquitination and degradation of AMPK in cancer, thereby regulating glucose metabolism in cancerous cells.^10, 14^ Our findings in this study suggest that decreased expression of FBP1 protein in HCC is mediated, at least partially through oncogenic MAGE-TRIM28 ubiquitin ligase-mediated protein degradation. Importantly, increased expression of TRIM28 associated with decreased expression of FBP1 and tumor node metastasis stage of HCC. Our results show that aberrant activation of MAGE proteins and overexpression of TRIM28 in HCC provide a novel mechanism to drive carcinogenesis in the liver and this effect can be attributed, at least in part, to their regulation of FBP1 protein degradation and the Warburg effect. Thus, our findings and those from other reports suggest that MAGE-TRIM28 ligase regulates multiple metabolic regulators (for example, AMPK and FBP1) to reprogram cancer cell metabolism.

As an effective proteasome inhibitor, bortezomib enables to suppress proliferation of HCC cells. Although bortezomib has not been effective for treatment of HCC as a single agent,^40^ it has been shown that bortezomib can improve the therapeutic effects when combined with other agents in HCC.^41^ Thus, the mechanisms of action of bortezomib in inhibiting HCC growth appear to be very complex. We demonstrated that bortezomib is capable of regulating the Warburg effect in HCC by inhibiting proteasome-dependent degradation of FBP1. Our findings suggest that inhibition of FBP1 degradation by bortezomib in combination with other drugs can be harnessed for effective treatment of HCC.

**Figure 7:**
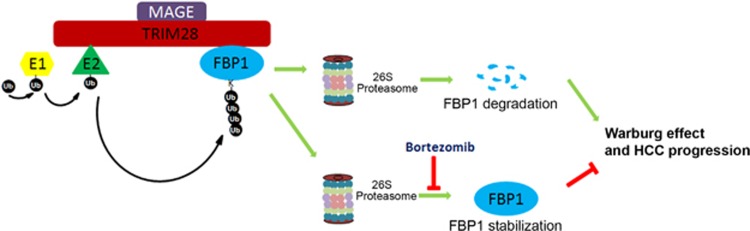
A hypothetical model depicting MAGE-TRIM28-mediated degradation of FBP1 and increased Warburg effect and cell growth in HCC cells overexpressing MAGE and TRIM28 proteins. However, this process can be inhibited by treatment of the proteasome inhibitor bortezomib and restoring the expression of FBP1 inhibits Warburg effect and blocks HCC progression.

In summary, we identify TRIM28 as an E3 ubiquitin ligase of FBP1.MAGE-A3 and MAGE-C2 can bind with TRIM28 to form MAGE-TRIM28 cancer-associated ubiquitin ligase in HCC. We discover that MAGE-A3 and MAGE-C2 can enhance the E3 ligase activity of TRIM28 in FBP1 degradation (Figure 7). Thus, our findings identify ubiquitination and proteasome degradation of FBP1 as a key mechanism contributing to downregulation of FBP1 proteins in HCC. Our study also demonstrates that MAGE-TRIM28 regulation of FBP1 degradation affects the Warburg effect and HCC progression, suggesting that MAGE-TRIM28 regulates reprogramming of cancer cell metabolism by causing protein degradation of multiple metabolic regulators including FBP1. Thus, inhibiting oncogenic MAGE-TRIM28 ubiquitin ligase-mediated degradation of substrates such as FBP1 could be a therapeutic option for the treatment of advanced HCC.

## Materials and methods

### Cell lines, cell transfection and treatment

All liver cancer cell lines, including SK-Hep-1 and HepG2, were recently purchased from ATCC (Manassas, VA, USA) and were authenticated via short tandem repeat profiling. HepG2 and SK-Hep-1 cells were propagated, respectively, in Dulbecco's modified Eagle's medium and Eagle's minimum essential medium (Thermo Fisher Scientific, Waltham, MA, USA) with 10% fetal bovine serum supplemented. All cell lines were routinely maintained at 37 ^o^C, 5% CO_2_, and passaged when cells became 90% confluent. Mycoplasma contamination was regularly examinedusing Lookout Mycoplasma PCR Detection Kit purchased from Sigma-Aldrich (St Louis, MO, USA). Transfections were conducted using lipid-based method (Lipofectamine 2000, Thermo Fisher Scientific) or by BTX ECM 830 Electroporation System (BTX, Holliston, MA, USA) following the manufacturer’s instructions. The efficiencies of transfection were about 75–90%. For glucose consumption measurement, both cell lines were maintained in Dulbecco's modified Eagle's medium without phenol red.

### Plasmids, antibodies and chemicals

Flag-tagged full-length FBP1 complementary DNA was subcloned into SBP vector. Expression vectors for HA-MAGE-A3, Myc-MAGE-A3, HA-MAGE-C2, Myc-MAGE-C2 were constructed using the pCMV plasmid. Bacterial expression vectors for GST-FBP1 and GST-TRIM28 recombinant proteins were generated using the pGEX-4T-1 backbone vector. His-FBP1 was subcloned into pET-28a (+) vector. Mammalian expression vector for HA-TRIM28 was purchased from Addgene (Cambridge, MA, USA). The KOD -Plus- Mutagenesis Kit (Toyobo, Osaka, Japan) was used to generate plasmids for HA-TRIM28-C65/68A, HA-TRIM28ΔRING and Myc-MAGE-C2-L161/162A. Information for antibodies used is provided in Supplementary Table S3. Chemicals used, including MG132, bortezomib and cycloheximide, were purchased from Sigma-Aldrich.

### Immunoprecipitation

Cells were harvested and resuspended in 1ml of RIPA buffer. After sonicated for 5 min, cell lysate was centrifuged for 15 min at 13 200 r.p.m. at 4 °C. The supernatant was transferred to a new tube and incubated with Pierce Protein G Agarose and primary antibody or IgG in the cold room overnight. The beads were washed five times with IP buffer, resuspended with sample loading buffer (Thermo Fisher Scientific) and heated at 100 °C for 5 min. The supernatant was used for further western blotting analysis.

### Western blotting

Cells were harvested and resuspended in RIPA buffer for 15 min on ice. Spin the cell lysate for 15 min at 13 200 r.p.m. at 4 °C. The supernatant were diluted in sample loading buffer (Thermo Fisher Scientific) and dithiothreitol and heated in 100 °C for 5  min. The samples were loaded on sodium dodecyl sulfate–polyacrylamide gel (7.5–10%) and run for 90  min at 100 V. Proteins were transferred from the gel to a nitrocellulose membrane. The membrane with protein of interest was placed in a box with 5% milk prepared in 1 × tris-buffered saline, 0.1% tween 20 (TBST) and the primary antibody in the appropriate dilution at 4 ^o^C overnight. Next day, the membrane was incubated with a horseradish peroxidase-conjugated secondary antibody and protein was detected by chemiluminescence.

### Tandem affinity purification

Briefly, SFB-tagged FBP1 was expressed in 293T cells. Cells were harvested and lysed in NETN buffer as described previously.^42^ Streptavidin sepharose beads (GE Healthcare Life Sciences, Pittsburgh, PA, USA) were added into the supernatant and gently rocked in cold room overnight. The beads were washed and eluted with 2mM of biotin. Subsequently, S-protein agarose beads (Novagen, Madison, WI, USA) were added into the elution products and gently rocked in cold room overnight. The beads were washed and subjected to sodium dodecyl sulfate–polyacrylamide gels for mass spectrometry and silver staining.

### Quantitative reverse transcriptase–PCR

Cellular RNA was isolated using TRIzol reagent purchased from Thermo Fisher Scientific. In all, 2 μg RNA was reversely transcribed by Superscript II reverse transcriptase (Thermo Fisher Scientific). Complementary DNA and gene-specific primerswere mixed with IQ SYRB Green Supermix for real-time (quantitative) PCR by using iCycler QTX detection system (Bio-Rad, Hercules, CA, USA). The 2-△Ct method was used to quantitate fold changes by normalizing to *GAPDH*. Primer for reverse transcriptase–quantitative PCR is provided in Supplementary Table S1.

### RNA interference

Lipofectamine 2000 were used to transfect 293T cells with shRNA plasmids (Sigma-Aldrich) and viral packaging plasmids (pVSV-G andpEXQV). Twenty-four hours after transfection, cell medium was replaced with Dulbecco's modified Eagle's medium supplemented 10% fetal bovine serum and 1:100 diluted sodium pyruvate. Forty-eight hours later, culture medium containing virus particles was collected and applied to liver cancer cells before treated with polybrene at the concentration of 12 μg/ml. Cells were collected 48 h after virus infection and puromycin selection. shRNA sequence information is provided in Supplementary Table S2.

### GST pull-down assay

Cultured liver cancer cells were harvested and cell lysate was prepared using lysis buffer (20 mM Tris-HCl (pH 7.5),150 mM NaCl, 0.1% Nonidet P-40, 1 mM dithiothreitol, 10% glycerol, 1 mM EDTA, 2.5 mM MgCl_2_ plus protease inhibitor cocktail) at cold room. Glutathione-sepharose beads (GE Healthcare Life Sciences, Pittsburgh, PA, USA) were used to capture GST-tagged recombinant proteins. Purified beads were subject to extensive wash and bound proteins were eluted in sample buffer, followed by sodium dodecyl sulfate–polyacrylamide gel electrophoresis and western blotting analysis.

### MTS assay

HepG2 or SK-Hep-1 cells were seeded in 96-well plates and incubated in cell incubator. The viability of cells was determined by MTS assay kit (Promega, Madison, WI, USA). Absorbance values (490 nm) were measured in a microplate reader.

### Measurement of glucose consumption and production of lactate

Liver cancer cells were plated in six-well plates and the culture medium were collected after incubation of cells for 48 h. The Glucose (GO) Assay kit (Sigma-Aldrich) and l-Lactate Assay kit (Eton Bioscience, San Diego, CA, USA) were then used to quantitatively measure the concentration of glucose and lactate in spent medium. Glucose consumption of liver cancer cells was obtained by using the formula: the glucose concentration of unused cell culture medium minus the glucose concentration in spent medium.

### Generation of HCC xenografts in mice

The mouse study was conducted according to the NIH guidelines and was approved by the IACUC at Mayo Clinic. Age-matched 6-week-old male NOD Scid interlukin-2 receptor gamma deleted mice were randomly separated into four groups (each group with four mice): shControl, shFBP1, shTRIM28 or both (shFBP1 plus shTRIM28). SK-Hep-1 cells (5 × 10^6^) infected with indicated lentivirus were suspended in 100 μl of 1:1 PBS/Matrigel (BD Biosciences, San Jose, CA, USA) and administered into subcutaneous layer of the right flank of mice. The caliper was used to determine the xenograft size every other day for 21 days. The xenograft volume was determined as length × width^2^/2. After 21-day observation, xenografts were harvested. Tumor tissues were divided for different purposes: a portion was fast-frozen into optimal cutting temperature compound (OCT) for frozen section, a portion was embedded by paraffin for IHC, and the rest was stored in −80 °C for protein and RNA isolation and western blotting and reverse transcriptase–quantitative PCR analyses.

### TMA and IHC scoring

The HCC TMA slides (Lot. HLivHCC150PG-01, US Biomax, Rockville, MD, USA), including 75 cases of normal tumor paired TMA specimens, were stained with FBP1 and TRIM28 antibodies by standard IHC procedures. The IHC staining was scored based on staining intensity and ratio of positive cells as described previously.^43^ A final staining index score for each specimen was determined using the following formula: ratio of positive cells × intensity.

### Statistical analysis

Pearson’s product-moment correlation was used to calculate the association between the staining index sore of FBP1 and TRIM28 in TMA slides. For comparing the difference between two groups, unpaired *t*-test (two trailed) was used. *P*=0.01 or smaller were considered statistically significant.
