# Supplementary material for: MAGE-TRIM28 complex promotes the Warburg effect and hepatocellular carcinoma progression by targeting FBP1 for degradation
Source: Oncogenesis. 2017 Apr 10;6(4):e312–. doi: 10.1038/oncsis.2017.21 (PMC5520498; doi:10.1038/oncsis.2017.21)
Supplement: Supplementary Information [file oncsis201721x1.docx]

**Supplementary Information**

**MAGE-TRIM28 complex promotes the Warburg effect and hepatocellular carcinoma progression by targeting FBP1 for degradation**

Xin Jin, Yunqian Pan, Liguo Wang, Lizhi Zhang, Ramya Ravichandran, Patrick Ryan Potts, Jingting Jiang, Heshui Wu and Haojie Huang

**Supplementary Table S1: Primers for RT-qPCR**

| Gene | Forward | Reverse |
| --- | --- | --- |
| *FBP1* | ACATCGATTGCCTTGTGTCC | CCACCAAAATGAACTCCCCG |
| *TRIM28* | TGTTTCCACCTGGACTGTCA | CCAGCAGTACACGCTCACAT |
| *GAPDH* | ACCCACTCCTCCACCTTTGAC | TGTTGCTGTAGCCAAATTCGTT |

**Supplementary Table S2: Sequences for shRNAs**

| shFBP1-1 | 5′-CCGGCCTTGATGGATCTTCCAACATCTCGAGATGTTGGAAGATCCATCAAGGTTTTTG-3′ |
| --- | --- |
| shFBP1-2 | 5′-CCGGCGACCTGGTTATGAACATGTTCTCGAGAACATGTTCATAACCAGGTCGTTTTTG-3′ |
| shTRIM28-1 | 5′-CCGGCCTGGCTCTGTTCTCTGTCCTCTCGAGAGGACAGAGAACAGAGCCAGGTTTTT-3′ |
| shTRIM28-2 | 5′-CCGGGAGAATTATTTCATGCGTGATCTCGAGATCACGCATGAAATAATTCTCTTTTT-3′ |
| shMAGE A3-1 | 5′-CCGGCCAGGTTTATGAATGACAGTACTCGAGTACTGTCATTCATAAACCTGGTTTTTTG-3′ |
| shMAGE A3-2 | 5′-CCGGCTGATAATCGTCCTGGCCATACTCGAGTATGGCCAGGACGATTATCAGTTTTTTG-3′ |
| shMAGE C2-1 | 5′-CCGGTTTATCTGGTTTAAGAGTAACCTCGAGGTTACTCTTAAACCAGATAAATTTTTTG-3′ |
| shMAGE C2-2 | 5′-CCGGAGTTAGCTTCAGAGTGTTAATCTCGAGATTAACACTCTGAAGCTAACTTTTTTTG-3′ |

**Supplementary Table S3: Antibodies**

| **Protein Name** | **Antibody category number** | **Dilution for WB** |
| --- | --- | --- |
| FBP1 | Abcam, ab109732 | 1:1,000 |
| TRIM28 | Abcam, ab10483 | 1:5,000 |
| MAGE-A3 | Abcam, ab140678 | 1:500 |
| MAGE-C2 | Santa Cruz Biotechnology, SC-68603 | 1:500 |
| ERK2 | Santa Cruz Biotechnology, SC-1647 | 1:5,000 |
| AMPKα | Abcam, ab32047 | 1:1,000 |
| Myc tag | Santa Cruz Biotechnology, SC-40 | 1:1,000 |
| Flag tag | M2, Sigma-Aldrich, F-3165 | 1:3,000 |
| HA tag | Covance, MMS-101R | 1:3,000 |

**Supplementary Figure Legends**

**Supplementary Figure S1**. (**a**) Increased expression of TRIM28 in HCC tissues (n=22) compared with the normal liver tissues (n=21) as revealed by Oncomining of the data reported by et al. ([Roessler et al 2010](#_ENREF_1)). (**b**) Western blot analysis of whole cell lysate, cytosolic, nuclear extracts and co-IP samples from HepG2 cells immunoprecipitated by IgG or anti-FBP1 antibody. Exp., exposure. (**c**) Western blot analysis of whole cell lysate, cytosolic, nuclear extracts and co-IP samples from HepG2 cells immunoprecipitated by IgG or anti-TRIM28 antibody. (**d**) HepG2 cells were transfected with expression vector or shRNAs as indicated. At 48 h after transfection, cells were harvested for RT-qPCR analysis of *TRIM28* and *FBP1* mRNAs. * *P* < 0.01; n.s., not significant. (**e**) Measurement of glucose consumption and L-lactate levels in the spent medium of HepG2 cells 48 h after infected with indicated constructs. SR, shRNA-resistant. * *P* < 0.01; n.s., not significant. (**f**) Western blot analysis of whole cell lysate from HeLa, HepG2 and SK-Hep1 cells. (**g**) HepG2 and SK-Hep-1 cells were infected with control or two independent TRIM28-specific shRNAs. 48 h after transfection, cells were harvested for western blot analysis.

**References in Supplementary Information**

Roessler S, Jia HL, Budhu A, Forgues M, Ye QH, Lee JS *et al* (2010). A unique metastasis gene signature enables prediction of tumor relapse in early-stage hepatocellular carcinoma patients. *Cancer Res* **70:** 10202-10212.
